# Contribution of multi-modal imaging to our understanding of dystonia pathogenesis

**DOI:** 10.1007/s00415-021-10696-2

**Published:** 2021-07-14

**Authors:** Claire MacIver, Kathryn Peall

**Affiliations:** 1 Neuroscience and Mental Health Research Institute, Cardiff University, Cardiff, UK; 2 CUBRIC, Cardiff University, Cardiff, UK

## Introduction

Dystonia is one of the most common forms of movement disorder, involving repetitive or sustained contractions of antagonistic muscle groups causing pain and abnormal posturing. The motor phenotypes observed in dystonia are varied with single muscle group involvement (focal), multiple adjacent (segmental) and more generalised forms involving multiple body regions. Some dystonia’s are task specific, being induced by undertaking a particular task or movement, whereas with others the involuntary movements and posturing are independent of activity. Dystonia is increasingly considered to be a network-based disorder involving multiple motor pathways in the brain, with the cerebellum, basal ganglia and sensorimotor cortex particularly implicated. Injected botulinum toxin represents the mainstay of treatment for many patients with focal and segmental forms and Deep Brain Stimulation (DBS) is used in the treatment of more severe forms.

While in excess of 30 disease-causing genes for dystonia have been identified, understanding of the pathophysiological mechanisms underpinning dystonia remains poor. Neuroimaging, whilst providing only indirect measures, is our most accessible tool in assessing in vivo brain structure and functioning in humans. The papers discussed below make use of a range of neuroimaging techniques to further investigate dystonia pathophysiology, with these including (i) structural imaging techniques to evaluate grey matter volume and white matter diffusion properties, (ii) resting-state functional MRI to compare regional and global motor network changes in isolated focal dystonia, (iii) positron emission tomography (PET) to glean insight into acetylcholine release in the DYT1 (*TorsinA*) genetic form of generalised dystonia.

### Brain structural changes in focal dystonia: what about task specificity? A multimodal MRI study

This study assessed for structural differences in patients with task-specific dystonia (TSD) compared to those with non-task-specific dystonia (NTSD). Three techniques were used to assess for subtle grey matter volumetric differences: (1) whole-brain cortical thickness analysis, an automated process which divides the cortex into subsections and measures for average cortical thickness; (2) Voxel-based morphometry (VBM), taking a voxel-by-voxel approach to compare localised brain volume changes; (3) Regional grey matter volume analysis. Changes to white matter structures were also assessed using Tract based Spatial Statistics (TBSS) of diffusion MR images, with this approach allowing for measurement of the diffusion properties within individual voxels.

A total of 97 patients with isolated focal dystonia and 83 age- and gender-matched controls were recruited. Amongst the dystonia cohort, 36 had TSD (21 writers’ cramp, 15 adductor spasmodic dysphonia) and 61 NTSD (27 blepharospasm, 34 cervical dystonia). Each patient was scanned at least 3 months after their last botulinum toxin injection, and in addition to MR scanning an extensive clinical assessment included dystonia severity rating, determination of dystonia triggers, pain severity and the presence of a sensory trick. Although matched for age and gender the clinical groups did differ with lower clinical severity and higher reported disability in those with TSD, while patients diagnosed with NTSD were more likely to have received botulinum toxin treatment and report pain.

Overall analysis found a significantly (*p* < 0.001) thicker primary somatosensory cortex (bilaterally) amongst those with TSD compared with both those with NTSD and unaffected controls, as well as greater thickening in multiple regions involved in somatosensory processing compared to NTSD, including the right paracentral, precentral gyri and inferior parietal gyri, and left superior parietal, rostral middle frontal, supramarginal, fusiform and inferior temporal gyri. In contrast, the NTSD cohort demonstrated cortical thinning compared to controls in a number of regions including the primary somatosensory cortex and premotor cortex. VBM measurements demonstrated similar changes, with increased grey matter volumes in TSD and decreased in NTSD in several regions involved in sensorimotor processing, with substantial overlap with those regions identified in the cortical thickness analysis. However, application of more stringent post-hoc corrections (Bonferroni Holm step down correction for multiple comparisons) these differences were lost with no between group statistically significant differences observed. The deep grey matter regional analysis demonstrated an increased volume in the left amygdala in TSD compared to both NTSD and healthy controls, and an increased volume of the right globus pallidus compared to NTSD. NTSD showed reduced thalamic volumes compared to healthy controls. White matter analysis showed widespread reductions in FA (fractional anisotropy—a measure of the directionality of the diffusion) and MD (mean diffusivity—the overall freedom of diffusion) in both groups compared to controls but more so in the TSD group.

*Comment *This study suggests potential mechanistic differences between TSD and NTSD, with findings indicating differences in diffusion characteristics as well as higher (TSD) and lower (NTSD) grey matter volumes in brain regions associated with sensorimotor processing compared to controls. The strengths of this study include the large recruited and matched cohort, as well as the use of multiple complimentary techniques in assessing brain structure. However, it is important to note that these techniques predominantly involved use of whole grey/white matter approaches rather than being driven by focusing on specific regions of interest. As a result, the data generated a large number of multiple comparisons, with statistically significant results only observed in the absence of correction for multiple comparisons.

*Tomić et al. (2020). Movement Disorders 36(1):196–205*

### Regional, not global, functional connectivity contributes to isolated focal dystonia

Functional MRI makes use of variation in the signal produced by oxygenated and deoxygenated blood due to differences in their magnetic properties (the BOLD signal-blood oxygen level-dependent signal) and is considered to be a proxy for neuronal activity. Resting state functional connectivity MRI measures changes in the BOLD signal in the brain at rest, and how that signal change correlates between brain regions. Norris et al. performed resting-state functional connectivity analyses in isolated focal dystonia, looking for shared changes between dystonia subtypes. They undertook two sets of analyses for each participant; (i) assessment of specific regional activity focussing on pre-determined regions in the basal ganglia and sensorimotor cortex, (ii) a more global analysis involving 300 regions across the whole brain. They recruited 58 patients diagnosed with isolated idiopathic focal dystonia [adductor laryngeal dystonia (*n* = 18), focal upper limb dystonia (*n* = 10), cervical dystonia (*n* = 13), craniofacial dystonia (*n* = 17)] and 47 age and gender-matched controls. Their intention was to undertake the imaging at least 3 months post the most recent botulinum toxin treatment, although this wasn’t achieved in all participants.

Following correction for multiple comparisons, no statistically significant between-group differences were observed using the global brain approach. However, on a regional level there was evidence of significantly weaker functional connectivity in those diagnosed with dystonia between the left posterodorsal putamen and the right striatum, as well as weaker negative functional connectivity between the bilateral sensorimotor mouth cortex and the left intraparietal sulcus.

*Comment *This study implicates regional differences in functional connectivity in focal dystonia between basal ganglia and sensorimotor regions, which is not associated with a more global disruption to functional connectivity. This potentially indicates some shared mechanisms between dystonia subtypes in key motor control regions, although the sizes of these individual subgroups were comparatively small and would benefit from replication in larger cohorts. However, this study employed substantial methodology aimed at reducing the impact of movement artifact, a potentially important confound in functional MR studies and of key importance in the investigation of a movement disorder population.

*Norris et al. (2020). Neurology 95:e2246–2258*

### Striatal and cerebellar vesicular acetylcholine transporter expression is disrupted in human DYT1 dystonia

Neurotransmitter activity is key in brain functioning, with disruption to acetylcholine neurotransmission having been implicated in animal models of dystonia. Mazere et al. applied PET techniques using a radioligand that binds to the vesicular acetylcholine transporter, involved in presynaptic acetylcholine storage and is a rate-limiting factor in the release of acetylcholine. The study focused on 17 brain regions involved in motor control or containing cholinergic cell bodies, with a particular focus on regions in the cerebellum and basal ganglia. In addition to the PET images, resting-state functional MR images were also captured, as well as volumetric structural analysis to enable adjustment for confounding volume loss that may contribute to changes in measured radioligand binding. Twenty individuals with genetic confirmation of a mutation in the *TorsinA* gene (DYT1 dystonia) were recruited, and compared to cohort of age- and gender-matched controls (*n* = 20). Three of those in the DYT1 cohort were prescribed routine anticholinergic medication at the time of the study, however, this was withheld for 72 h prior to scanning.

In comparison with controls, those with DYT1 mutations demonstrated significantly lower levels of vesicular acetylcholine transporter binding in the posterior putamen. This difference also appeared to be age dependent, only present in the younger group when the cohort was split into two groups above and below the overall median age of the cohort. Reduced binding was also observed in the cerebellum of the patient cohort, most notably the vermis, with no age effect seen in this region. The functional MR analysis once again demonstrated higher between region connectivity in those diagnosed with dystonia, as well as being positively correlated with ligand binding in the same regions, a finding not observed amongst the control cohort. The volumetric analysis only showed between-group differences in the right caudate and right dorsoventrolateral prefrontal cortex, with higher volumes in patients. There was an age effect on volume, but no age by group interaction.

*Comment* This study implicates disruption to cholinergic neurotransmission in the basal ganglia and cerebellum in DYT1 dystonia. The study benefits from its focussed, region of interest-driven analysis, rather than a more global whole-brain approach. The additional volumetric analysis also considerably strengthened this study, ensuring adjustment for potentially confounds of systematic volumetric differences. Finally, the inclusion of only those with confirmed DYT1 mutations also allowed for greater homogeneity in assumed underlying pathogenesis, in spite of there being considerable clinical phenotypic heterogeneity.

*Mazere et al. (2021). Brain 144(3):909–923*

